# Deep learning-based automated masseter muscle area on routine CT stratifies survival in oral cancer

**DOI:** 10.1007/s11282-026-00923-9

**Published:** 2026-04-11

**Authors:** Shin-ichiro Hiraoka, Katsuya Sakamoto, Kohei Kawamura, Shuji Uchida, Ryo Akiyama, Susumu Tanaka

**Affiliations:** Department of Oral and Maxillofacial Surgery, Graduate School of Dentistry, The University of Osaka, 1-8 Yamada-Oka, Suita, Osaka 565-0871 Japan

**Keywords:** Oral cancer, Oral squamous cell carcinoma, Masseter muscle, Sarcopenia, Deep learning, Computed tomography

## Abstract

**Objectives:**

To develop and validate a deep learning model for automated masseter muscle segmentation on routine head and neck CT and to evaluate whether the derived masseter muscle area is associated with overall survival in oral cancer.

**Materials and methods:**

A U-Net-based model was trained using a model-development cohort (n = 348) with preoperative CT and bioelectrical impedance analysis. Masseter muscle area was measured on an axial slice at the maxillary sinus floor, and sex-specific cutoffs for low masseter muscle area were derived against sarcopenia defined by appendicular skeletal muscle index. Prognostic value of AI-derived masseter muscle area (AI-MMA) was tested in an independent cohort of primary oral cancer patients (n = 247) using Kaplan–Meier analysis and Cox proportional hazards models.

**Results:**

Segmentation performance was high (Dice similarity coefficient, 0.92). AI-MMA correlated strongly with manual MMA in males and females (r = 0.892 and r = 0.896, respectively; both p < 0.001). Low AI-MMA was associated with poorer overall survival. In multivariable analysis, low AI-MMA remained an independent predictor of mortality (hazard ratio [HR], 2.584; 95% CI, 1.132–5.898; p = 0.024), together with stage III–IV disease (HR, 5.811; 95% CI, 2.130–15.860; p < 0.001) and low body mass index (HR, 2.572; 95% CI, 1.162–5.693; p = 0.020).

**Conclusions:**

Automated AI-MMA from routine staging CT provides an objective prognostic biomarker in oral cancer.

## Introduction

Oral cancer, most commonly oral squamous cell carcinoma (OSCC), remains a major global health problem with persistently high mortality despite advances in multimodal treatment [1, 2]. Several countries have reported increasing incidence, driven in part by population aging and continued exposure to established risk factors, and registry-based data from Japan likewise document a substantial disease burden of head and neck cancer including oral cancer [1–3]. Because outcomes vary widely even among patients with similar clinicopathological characteristics, clinicians need objective and readily available prognostic markers to support pretreatment risk stratification and treatment planning [4]. Against this background, there is growing interest in markers that reflect patients’ nutritional and functional reserve, yet scalable approaches that can be applied reliably to routine head-and-neck CT remain limited.

In recent years, body composition has been recognized as a crucial determinant of clinical outcomes in oncology. Sarcopenia, a syndrome characterized by a progressive and generalized loss of skeletal muscle mass and function, is now established as a powerful independent predictor of postoperative complications, treatment toxicity, and poor survival across various cancer types [5, 6]. Patients with head and neck cancer are particularly susceptible to sarcopenia due to factors such as tumor-induced dysphagia, cancer cachexia, and the metabolic demands of the disease and its treatment [7, 8]. Therefore, the objective assessment of muscle mass is a critical component of pretreatment evaluation.

Computed tomography (CT) imaging, routinely performed for cancer staging, offers a reliable and opportunistic method for quantifying muscle mass. The cross-sectional skeletal muscle area at the third lumbar vertebra (L3) is commonly used in oncology studies as an imaging-based surrogate for systemic muscle mass [9]. However, this method is often impractical for oral cancer patients, as their routine diagnostic imaging is typically limited to the head and neck region, and abdominal CT scans are not standardly acquired [10]. This has spurred the search for alternative, anatomically relevant muscle groups within the head and neck scan range.

The masseter muscle has emerged as a viable surrogate for assessing systemic muscle status in this patient population [11]. It is consistently visualized on standard head and neck CT scans, and its measurement is less susceptible to artifacts from dental restorations compared to other regional muscles. Several studies have demonstrated a significant association between a reduced masseter muscle volume or area and poor prognosis in patients with head and neck, esophageal, and other cancers [12, 13]. However, the clinical utility of this measurement is hampered by the reliance on manual segmentation, a process that is labor-intensive, time-consuming, and subject to significant inter- and intra-observer variability, limiting its widespread adoption in busy clinical workflows [14].

Artificial intelligence (AI), particularly deep learning, has shown transformative potential in medical image analysis, offering solutions to automate complex and repetitive tasks [15, 16]. Convolutional neural networks (CNNs), and specifically the U-Net architecture, have become the de facto standard for biomedical image segmentation, demonstrating human-level performance in various tasks, including organ and tumor delineation [17, 18]. By automating the segmentation process, deep learning can provide rapid, objective, and reproducible measurements, overcoming the key limitations of manual methods [19, 20]. While some studies have explored deep learning for masseter muscle segmentation, they have often been limited to smaller datasets or have not validated the clinical prognostic value of the automated measurements [21, 22].

Therefore, this study had two primary objectives: first, to develop and validate a robust deep learning model based on the U-Net architecture for the fully automated segmentation of the masseter muscle cross-sectional area on routine pretreatment CT scans. Second, to investigate the clinical utility of this automated measurement as an independent prognostic factor for overall survival in a large, well-characterized cohort of oral cancer patients. We hypothesized that an automatically quantified, low masseter muscle area would be a significant predictor of poor prognosis, and that our AI-driven tool could facilitate objective risk stratification in this patient population.

## Materials and methods

### Study design and patient cohorts

This retrospective cohort study was conducted in two parts, utilizing two distinct patient cohorts from the Department of Oral and Maxillofacial Surgery at Osaka University Dental Hospital. The study protocol was approved by the Institutional Review Board of Osaka University Dental Hospital (Approval No. H29-E19) and was conducted in accordance with the Declaration of Helsinki. The requirement for written informed consent was waived by the ethics committee due to the retrospective nature of the analysis.

First, a model development cohort of 348 patients who underwent surgery under general anesthesia between January 2017 and December 2020 was used to train and validate the deep learning model. These patients had undergone preoperative head and neck CT imaging and bioelectrical impedance analysis (BIA). Exclusion criteria were age < 20 years, presence of infections that could affect nutritional status, and a history of head and neck dysplasia. This cohort was used to establish the ground truth for muscle segmentation and to determine the optimal cutoff values for sarcopenia based on muscle area. The characteristics of the model development cohort are summarized in Table 1.

**Table 1:** The data for setting the cutoff values

Characteristics	n = 348	
	n	ratio (%)
Sex (male/female)	177/171	50.9/49.1
Age (years)		
mean ± SD	41.0 ± 20.2	
median (range)	33 (20–89)	
Case		
Jaw deformity	181	52
Malignant tumor	79	22.7
Cyst	35	10.1
Cleft lip and palate	16	4.6
Benign tumor	15	4.3
Fracture	11	3.1
Leukoplakia	3	0.9
Sialolith	3	0.9
Others	5	1.4
CRP (mg/dL)	0.24 ± 0.47	
Alb (g/dL)	4.50 ± 0.36	
A/G	1.63 ± 0.28	
TC (mg/dL)	190.87 ± 32.14	
BMI (kg/m^2^)	21.79 ± 3.60	
CAR	0.06 ± 0.12	
NLR	2.29 ± 1.28	
PLR	155.13 ± 69.47	
PNI	53.92 ± 4.87	
mGPS (0/1/2)	331/14/3	95.1/4/0.9
CONUT (0/1/2/3/4/6)	95/145/77/24/6/1	27.3/41.7/22.1/6.9/1.7/0.3
SMI (kg/m^2^)		
male	7.62 ± 0.85	
female	5.88 ± 0.72	
MMA (mm^2^)		
male	965.79 ± 200.95	
female	690.45 ± 151.65	

Others = impacted wisdom tooth (n = 1), temporomandibular joint ankylosis (n = 1), unreduced dislocation of the temporomandibular joint (n = 1), residual fistula (n = 1), foreign body on the oral floor (n = 1)

*CAR* CRP/albumin ratio, *NLR* neutrophil/lymphocyte ratio, *PLR* platelet-to-lymphocyte ratio, *PNI* prognostic nutrition index, *mGPS* modified Glasgow prognostic score, *CONUT* controlling nutritional status, *SMI* skeletal muscle index, *MMA* masseter muscle cross-sectional area

Second, a separate prognostic validation cohort of 247 patients with primary oral cancer who received treatment between January 2009 and December 2020 was used to evaluate the clinical and prognostic significance of the automated measurements. All patients had preoperative head and neck CT scans. Exclusion criteria for this cohort were direct tumor invasion into the masseter muscle, prior treatment for oral cancer, and age < 20 years, to ensure that measurements were not confounded by tumor presence or developmental changes. The characteristics of the prognostic validation cohort are summarized in Table 2.

**Table 2:** Validation data

Characteristics	n = 247	
	n	ratio (%)
Sex (male/female)	139/108	56.3/43.7
Age (years)		
mean ± SD	63.2 ± 15.5	
median (range)	66 (20–90)	
Tumor location		
Tongue/Gingival/Oral floor/Buccal	99/77/27/17	40.1/31.2/10.9/6.9
Palate/Intraosseous/Maxillary sinus	11/7/4	4.5/2.8/1.6
Lip/Submandibular gland/Sublingual gland	3/1/1	1.2/0.4/0.4
Stage		
Ⅰ/Ⅱ/Ⅲ/Ⅳ	85/65/16/81	34.4/26.3/6.5/32.8
Treatment		
Operation (Ope)	172	69.6
Chemotherapy (Chemo)	2	0.8
Radiation therapy (RT)	2	0.8
Ope + Chemo/RT	60	24.3
Chemo + RT	11	4.5
CRP (mg/dL)	0.40 ± 0.58	
Alb (g/dL)	4.30 ± 0.43	
A/G	1.48 ± 0.30	
T-cho (mg/dL)	198.39 ± 37.42	
BMI (kg/m^2^)	21.22 ± 3.46	
CAR	0.10 ± 0.16	
NLR	2.53 ± 1.36	
PLR	154.65 ± 67.35	
PNI	51.37 ± 5.69	
mGPS (0/1/2)	218/24/5	88.3/9.7/2.0
CONUT (0/1/2/3/4/6/7)	62/93/54/26/10/1/1	25.1/37.7/21.9/10.5/4.0/0.4/0.4
MMA (mm^2^)		
male	993.75 ± 244.46	
female	762.62 ± 177.28	

Palate/Intraosseous/: treated intraosseous carcinoma as a gingival carcinoma

Stage: according to the TNM classification UICC 8th edition

*CAR* CRP/albumin ratio, *NLR* neutrophil/lymphocyte ratio, *PLR* platelet-to-lymphocyte ratio, *PNI* prognostic nutrition index, *mGPS* modified Glasgow prognostic score, *CONUT* controlling nutritional status, *MMA* masseter muscle cross-sectional area

### CT image acquisition and ground truth segmentation

Pre-treatment head and neck CT scans were acquired using a multi-detector CT scanner. Imaging parameters were standardized as follows: 120 kVp, 200–330 mA, and slice thickness of 2.5–5.0 mm. Both contrast-enhanced and non-contrast scans were included.

For all patients in the model development cohort (n = 348), the ground truth segmentation of the masseter muscle was performed manually. The cross-sectional area of both the left and right masseter muscles was delineated at the axial slice corresponding to the floor of the maxillary sinus. This anatomical level was selected as a readily identifiable landmark to facilitate reproducible slice selection; additionally, when feasible, this level may reduce the influence of dental metal artifacts compared with more caudal mandibular levels. CT-based cross-sectional area of the jaw muscles has been shown to correlate with their physiological cross-sectional area [23]. The segmentation was performed by a trained oral and maxillofacial surgeon using Synapse VINCENT® v5.3 (FUJIFILM, Tokyo, Japan). To ensure accuracy and minimize bias, all manual segmentations were reviewed and confirmed by a board-certified oral and maxillofacial radiologist with over 10 years of experience.

### Deep learning model for automated segmentation

#### U-Net architecture

We developed our automated segmentation model based on the U-Net architecture, a CNN renowned for its efficacy in biomedical image segmentation [17]. The U-Net consists of a contracting path (encoder) to capture contextual information and a symmetric expanding path (decoder) to enable precise localization. Our implementation was based on the nnU-Net framework, a robust, self-configuring version of U-Net that adapts its architecture and hyperparameters to the specific dataset, ensuring optimal performance [18]. The model architecture included 19 convolutional layers with Rectified Linear Unit (ReLU) activation, four max-pooling layers for downsampling, and corresponding up-sampling layers in the decoder path. The input images were resized to 256 × 256 pixels for model training.

#### Model training and validation

The model development cohort (n = 348) was randomly partitioned into training (80%, n = 278), validation (10%, n = 35), and internal testing (10%, n = 35) sets. To enhance the model’s robustness and prevent overfitting, we employed an extensive data augmentation strategy, including random rotations (± 15 degrees), scaling (± 10%), elastic deformations, and horizontal flipping.

The model was trained for 100 epochs with a batch size of 32. We used the Adam optimizer with an initial learning rate of 0.001, which was adaptively reduced on a plateau of the validation loss. A combination of Dice loss and binary cross-entropy was used as the loss function to handle the class imbalance and optimize for segmentation overlap. Training was performed on an NVIDIA Tesla V100 GPU with 32 GB of memory, using the PyTorch framework.

### Measurement of muscle area and nutritional indices

The total masseter muscle cross-sectional area (MMA) was calculated as the sum of the areas of the left and right masseter muscles. For the automated method, the area was calculated from the segmentation masks generated by our trained U-Net model (termed AI-MMA) using ImageJ software (v1.53, National Institutes of Health, USA).

In the model development cohort, the skeletal muscle index (SMI) was measured using an InBody570™ BIA device (InBody Japan, Tokyo, Japan) to serve as a reference for systemic muscle mass. The MMA cutoff values for sarcopenia were determined using receiver operating characteristic (ROC) curve analysis, with the established SMI diagnostic criteria from the Asian Working Group for Sarcopenia (AWGS 2019) as the reference standard (< 7.0 kg/m^2^ for males, < 5.7 kg/m^2^ for females) [24].

In the prognostic validation cohort, we collected data on various nutrition-related factors, including C-reactive protein (CRP), albumin (Alb), body mass index (BMI), and several prognostic scores such as the CRP/Alb ratio (CAR), neutrophil/lymphocyte ratio (NLR), and the Controlling Nutritional Status (CONUT) score [25, 26].

### Statistical analysis

All statistical analyses were performed using EZR (Saitama Medical Center, Jichi Medical University, Japan), a graphical user interface for R (The R Foundation for Statistical Computing). A p-value < 0.05 was considered statistically significant.

The segmentation performance of the deep learning model was evaluated on the internal test set by comparing the AI-MMA masks to the manual ground truth masks using the Dice Similarity Coefficient (DSC). The correlation between AI-MMA and manually measured MMA was assessed using Pearson’s correlation coefficient and visualized with a Bland–Altman plot.

Overall survival (OS), defined as the time from primary treatment to death from any cause, was the primary endpoint. The prognostic significance of low AI-MMA was assessed in the validation cohort. Kaplan–Meier curves were generated to visualize survival differences, and the log-rank test was used for comparison. Univariate and multivariate Cox proportional hazards regression models were used to identify independent prognostic factors for OS. Variables with a p-value < 0.10 in the univariate analysis, along with clinically relevant factors such as age, sex, and tumor stage (UICC 8th edition), were included in the multivariate model.

### Ethical statements

The study was conducted in accordance with the Declaration of Helsinki and approved by the Institutional Review Board of Osaka University Dental Hospital (protocol code H29-E19). Patient consent was waived for this retrospective analysis. All patient data were fully anonymized prior to analysis to protect confidentiality.

### Data availability

The datasets generated and/or analyzed during the current study are not publicly available due to privacy and ethical restrictions concerning patient confidentiality but are available from the corresponding author on reasonable request.

## Results

### Patient characteristics

The prognostic validation cohort comprised 247 oral cancer patients (139 males and 108 females) with a mean age of 63.2 ± 15.5 years (median, 66; range, 20–90). The most common primary site was the tongue (40.1%), followed by the gingiva (31.2%), oral floor (10.9%), and buccal mucosa (6.9%). According to the UICC 8th edition staging, 60.7% of patients had stage I–II disease and 39.3% had stage III–IV disease. Detailed patient demographics and clinical characteristics are summarized in Table 2.

### Deep learning model performance and MMA cutoff values

**Fig. 1:**
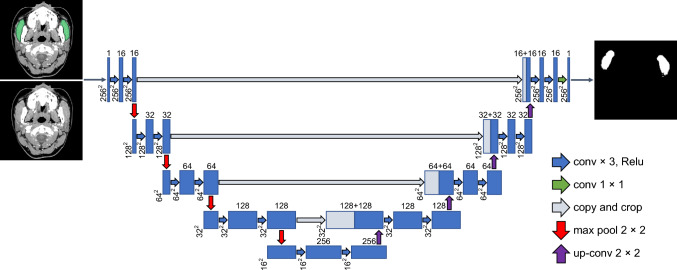
U-Net architecture for automated segmentation of the bilateral masseter muscles on an axial head-and-neck CT slice. The network uses an encoder–decoder structure with skip connections and outputs a binary mask of the masseter muscles for automated area quantification

On the internal test set (n = 35), our U-Net model demonstrated excellent performance for automated masseter muscle segmentation, and the network architecture is illustrated in Fig. 1. The mean Dice Similarity Coefficient (DSC) between the AI-generated masks and the manual ground truth was 0.92 ± 0.04, indicating a high degree of spatial overlap. The segmentation process was highly efficient, with the model taking less than 1 s per CT slice, a significant reduction from the 5–10 min required for manual segmentation.

**Fig. 2:**
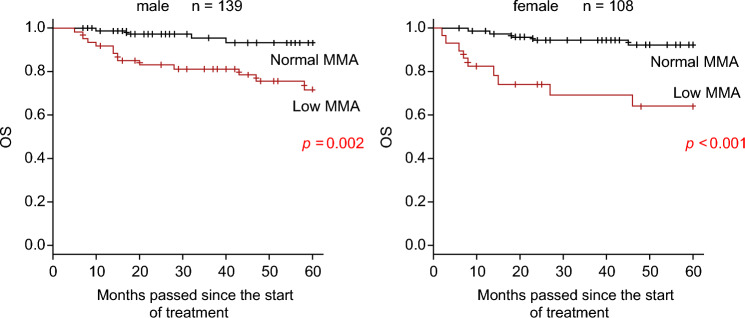
Kaplan–Meier overall survival curves stratified by manually measured masseter muscle area (MMA) using sex-specific cutoffs. The left panel shows males (n = 139) and the right panel shows females (n = 108). P values shown in the plots are from log-rank tests

Using the model development cohort (n = 348), ROC analysis determined the optimal MMA cutoff values for predicting sarcopenia (as defined by AWGS BIA criteria). The cutoff value for males was 926.97 mm^2^ (AUC = 0.696), and for females, it was 631.81 mm^2^ (AUC = 0.690). These thresholds were used to stratify patients in the validation cohort into low MMA and normal MMA groups (Fig. 2).

### Correlation between automated and manual measurements

**Fig. 3:**
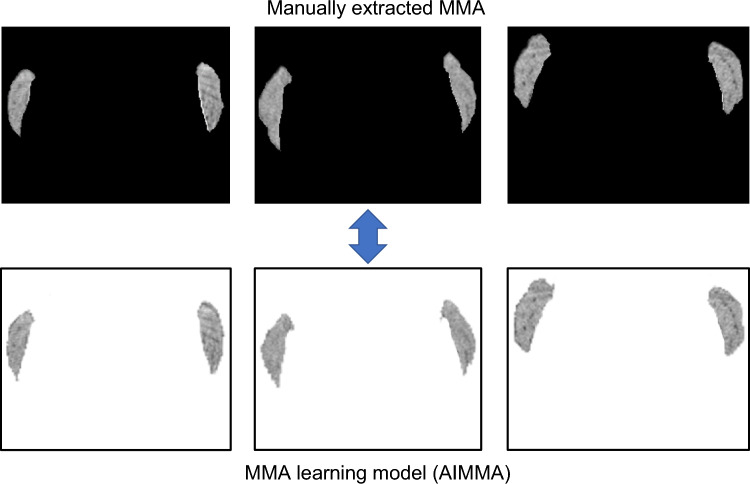
Representative examples comparing manual and automated masseter muscle segmentations. The top row shows manual segmentations used to derive MMA, and the bottom row shows automated segmentations generated by the AIMMA model, demonstrating close visual agreement

**Fig. 4:**
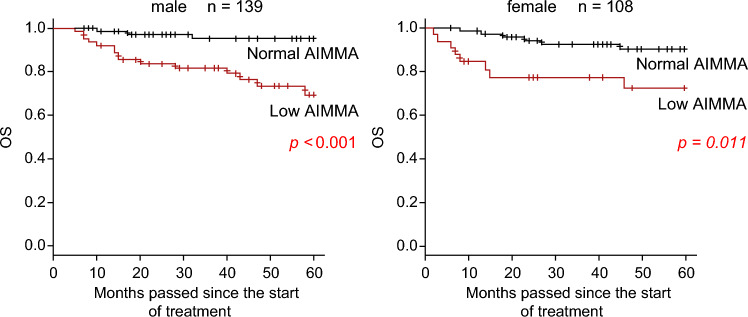
Kaplan–Meier overall survival curves stratified by automatically measured masseter muscle area (AI-MMA) derived from the AIMMA model using sex-specific cutoffs. The left panel shows males (n = 139) and the right panel shows females (n = 108). In the plots, the labels “Normal AIMMA” and “Low AIMMA” indicate normal and low AI-MMA, respectively; P values shown are from log-rank tests

In the prognostic validation cohort (n = 247), the automatically measured MMA (AI-MMA; denoted as “AIMMA” in Figs. 3 and 4) showed a very strong and highly significant positive correlation with the manually measured MMA. The Pearson correlation coefficient (r) was 0.892 for males (p < 0.001) and 0.896 for females (p < 0.001). Representative examples of manual and automated segmentations are shown in Fig. 3, illustrating close visual agreement between the two approaches.

### Prognostic significance of automated MMA (AI-MMA)

Using the established cutoffs, 38.9% of male patients and 35.2% of female patients in the validation cohort were classified as having low AI-MMA. Kaplan–Meier analysis demonstrated significantly poorer OS for patients with low AI-MMA in both males (p < 0.001) and females (p = 0.011) (Fig. 4). The 5-year OS rate was 58.3% in the low AI-MMA group, versus 82.1% in the normal AI-MMA group (Table 3).

**Table 3:** Univariate and multivariate Cox proportional hazards analyses for overall survival (manual MMA)

Variables		Univariate analysis (Cox proportional hazards model)		Multivariate analysis (COX proportional hazards model)	
	cutoff	HR	*p*-value	HR	*p*-value
		(95% CI)		(95% CI)	
Age	77	1.502	0.342		
		(0.542–3.787)			
Sex	male or female	0.999	1.000		
		(0.443–2.292)			
Stage	Ⅰ･Ⅱ or Ⅲ･Ⅳ	10.844	< 0.001	5.664	< 0.001
		(3.897–37.593)		(2.075–15.460)	
CRP (mg/dL)	1.15	3.410	0.041	0.526	0.394
		(0.862–11.690)		(0.120–2.308)	
Alb (g/dL)	4.3	1.696	0.181		
		(0.752–3.863)			
A/G	1.58	2.715	0.031	1.218	0.700
		(1.035–8.414)		(0.446–3.325)	
T-cho (mg/dL)	143	0.599	1.000		
		(0.013–4.395)			
mGPS	2	10.818	0.017	3.065	0.232
		(1.188–134.771)		(0.489—19.190)	
CONUT	2	4.520	< 0.001	1.978	0.118
		(1.790–11.149)		(0.842—4.650)	
CAR	0.033	2.399	0.123		
		(0.788–9.835)			
NLR	2.398	3.206	0.003	1.260	0.605
		(1.393–7.772)		(0.525–3.022)	
PLR	189.51	1.867	0.123		
		(0.756–4.399)			
PNI	53.888	3.366	0.022	1.144	0.831
		(1.115–13.715)		(0.330–3.966)	
BMI (kg/m^2^)	20.233	4.372	< 0.001	2.484	0.025
		(1.839–11.295)		(1.122–5.502)	
MMA (mm^2^)	male 926.970	5.602	< 0.001	2.839	0.012
	female 631.810	(2.346–14.527)		(1.256–6.415)	

Low MMA, the tumor stage, and BMI were poor independent prognostic factors (HR: 2.839 [95% CI, 1.256–6.415]/*p* = 0.012)

In univariate Cox analyses, advanced tumor stage and several inflammation- and nutrition-related indices were associated with OS, and low AI-MMA was also significantly associated with poorer OS (Table 4). In multivariate Cox proportional hazards modeling, low AI-MMA remained independently associated with poorer OS (HR, 2.584; 95% CI, 1.132–5.898; p = 0.024), together with stage III–IV disease (HR, 5.811; 95% CI, 2.130–15.860; p < 0.001) and low BMI (HR, 2.572; 95% CI, 1.162–5.693; p = 0.020) (Table 4).

**Table 4:** Univariate and multivariate Cox proportional hazards analyses for overall survival (AI-MMA)

Variables		Univariate analysis (Cox proportional hazards model)		Multivariate analysis (COX proportional hazards model)	
	cutoff	HR	*p*-value	HR	*p*-value
		(95% CI)		(95% CI)	
Age	77	1.502	0.342		
		(0.542–3.787)			
Sex	male or female	0.999	1.000		
		(0.443–2.292)			
Stage	Ⅰ･Ⅱ or Ⅲ･Ⅳ	10.844	< 0.001	5.811	< 0.001
		(3.897–37.593)		(2.130–15.860)	
CRP (mg/dL)	1.15	3.410	0.041	0.528	0.397
		(0.862–11.690)		(0.121–2.313)	
Alb (g/dL)	4.3	1.696	0.181		
		(0.752–3.863)			
A/G	1.58	2.715	0.031	1.132	0.809
		(1.035–8.414)		(0.414–3.096)	
T-cho (mg/dL)	143	0.599	1.000		
		(0.013–4.395)			
mGPS	2	10.818	0.017	3.129	0.225
		(1.188–134.771)		(0.496–19.750)	
CONUT	2	4.520	< 0.001	2.093	0.088
		(1.790–11.149)		(0.896–4.890)	
CAR	0.033	2.399	0.123		
		(0.788–9.835)			
NLR	2.398	3.206	0.003	1.168	0.731
		(1.393–7.772)		(0.482–2.827)	
PLR	189.507	1.867	0.123		
		(0.756–4.399)			
PNI	53.888	3.366	0.022	1.184	0.791
		(1.115–13.715)		(0.340–4.127)	
BMI (kg/m^2^)	20.233	4.372	< 0.001	2.572	0.020
		(1.839–11.295)		(1.162–5.693)	
AIMMA (mm^2^)	male 926.970	4.937	< 0.001	2.584	0.024
	female 631.810	(2.072–12.777)		(1.132–5.898)	

Low AIMMA, the tumor stage, and BMI were poor independent prognostic factors (HR: 2.584, 95% CI: 1.132–5.898, *p* = 0.024)

For comparison, in the model using manually measured MMA, low MMA also remained independently associated with poorer OS (HR, 2.839; 95% CI, 1.256–6.415; p = 0.012), together with stage III–IV disease (HR, 5.664; 95% CI, 2.075–15.460; p < 0.001) and low BMI (HR, 2.484; 95% CI, 1.122–5.502; p = 0.025) (Table 3).

## Discussion

In this study, we developed and validated a deep learning model for automated masseter muscle segmentation on routine head and neck CT scans (Fig. 1). Our key findings are twofold. First, the model achieved high segmentation performance and produced automated measurements that closely aligned with manual assessment, as illustrated by representative manual and automated segmentations (Fig. 3). Second, the automatically quantified masseter muscle area (AI-MMA) provided clinically meaningful prognostic stratification, with survival differences observed when MMA was assessed manually (Fig. 2) and when it was assessed automatically (Fig. 4). To our knowledge, this is one of the largest studies to not only develop an automated segmentation tool but also to rigorously validate its clinical prognostic value in an independent patient cohort.

The imperative for objective, reproducible, and efficient prognostic biomarkers in oncology is clear. Sarcopenia has been consistently linked to adverse outcomes, yet its integration into routine clinical practice has been slow, largely due to the logistical challenges of measurement [27]. Our AI-driven approach directly addresses this implementation gap. By automating the measurement of a clinically relevant muscle group from existing diagnostic images, our tool provides a practical solution for sarcopenia assessment that requires no additional procedures, costs, or significant clinician time. The model’s high performance (DSC of 0.92) is comparable to or exceeds that reported in other medical image segmentation tasks and is a testament to the power of the nnU-Net framework and our robust training methodology [18, 28].

Our clinical findings confirm the prognostic importance of muscle mass in oral cancer. In multivariate analysis, low AI-MMA remained independently associated with poorer OS (HR, 2.584; 95% CI, 1.132–5.898; p = 0.024), together with advanced tumor stage and low BMI, supporting the clinical relevance of this CT-derived biomarker beyond conventional staging. This finding aligns with a growing body of evidence linking sarcopenia to adverse outcomes in head and neck cancer [11, 29]. Patients with lower muscle mass may have diminished physiological reserves, rendering them more vulnerable to the catabolic stresses of cancer and its treatment, leading to higher rates of complications and reduced survival [30]. By providing an objective measure of this vulnerability, our tool can help clinicians identify high-risk patients who may benefit from targeted interventions, such as preoperative nutritional support, personalized physical therapy, or modified treatment regimens.

Compared with our previous study demonstrating the prognostic value of manually measured masseter volume [13], the current work represents a significant advancement. This study automates the process using a standardized 2D cross-sectional area at the maxillary sinus floor, supported by representative manual and automated segmentations (Fig. 3), and it validates the prognostic utility of the resulting measurement through survival stratification (Figs. 2 and 4). The use of a 2D area at a standardized anatomical landmark simplifies the workflow and may be more robust to variations in scan coverage compared to 3D volume measurement, thereby improving clinical applicability.

Our study has several strengths, including the use of a large and well-defined patient cohort, the development of a robust deep learning model using a state-of-the-art framework, and the rigorous validation of the automated measurement’s prognostic significance in a separate cohort. However, we also acknowledge several limitations. First, this was a retrospective study conducted at a single institution, which may limit the generalizability of our findings. Future work should involve external validation using multi-center, multi-vendor datasets to ensure the model’s robustness. Second, our analysis focused on muscle area as a surrogate for mass and did not incorporate additional CT-derived body composition metrics, such as muscle radiodensity or intramuscular fat infiltration (myosteatosis). Such factors may be relevant to clinically important outcomes in head and neck cancer, including postoperative wound complications, and warrant further study [31]. Future models could be trained to segment and quantify both muscle and fat tissue. Third, while we used the AWGS criteria for BIA-defined sarcopenia to establish our MMA cutoffs, the correlation was moderate (AUC ~ 0.70), suggesting that masseter muscle area and systemic SMI capture related but not identical aspects of body composition. Finally, our study focused on overall survival; further research is needed to explore the association of AI-MMA with other important outcomes, such as disease-free survival, treatment response, and postoperative complications.

In conclusion, our deep learning-based tool for automated masseter muscle segmentation is accurate, efficient, and clinically significant. The automatically quantified muscle area is a strong, independent predictor of survival in oral cancer patients. This work paves the way for the integration of automated body composition analysis into the standard oncologic workflow, providing clinicians with a powerful new tool for risk stratification and personalized patient management. Future prospective studies are warranted to confirm the clinical impact of integrating this AI-driven biomarker into treatment decision-making.

## Conclusions

We have successfully developed and validated a deep learning model that provides a fast, accurate, and fully automated method for measuring the masseter muscle cross-sectional area from routine head and neck CT scans. The automatically quantified muscle area proved to be a robust and independent predictor of overall survival in patients with oral cancer. This AI-powered tool offers a practical and objective means for assessing sarcopenia, enabling improved prognostic stratification and potentially guiding personalized therapeutic strategies in the clinical management of oral cancer.

## Data Availability

The datasets generated and/or analyzed during the current study are not publicly available due to privacy and ethical restrictions concerning patient confidentiality but are available from the corresponding author on reasonable request.
